# Dirubidium digallium oxide bis­(ortho­borate)

**DOI:** 10.1107/S1600536808005783

**Published:** 2008-03-07

**Authors:** Robert W. Smith, Chunhua Hu, Christopher D. DeSpain

**Affiliations:** a Department of Chemistry, University of Nebraska at Omaha, Omaha, NE 68182-0109, USA; b Nebraska Center for Materials and Nanoscience, University of Nebraska, Lincoln, NE 68588-0304, USA

## Abstract

The title compound, Rb_2_Ga_2_O(BO_3_)_2_, is part of the homologous series *A*_2_Ga_2_O(BO_3_)_2_ (*A* = Na, K, Rb and Cs). The structure contains pairs of gallium-centered tetra­hedra connected through a shared oxygen vertex. Orthoborate triangles connect the basal vertices of the tetra­hedra, forming a three-dimensional network with voids occupied by rubidium ions.

## Related literature

For related literature, see: Chen *et al.* (2004 ▶); Corbel & Leblanc (2000 ▶); Smith (1995 ▶, 1997 ▶).

## Experimental

### 

#### Crystal data

Rb_2_Ga_2_O(BO_3_)_2_
*M* *_r_* = 444.00
Monoclinic, 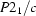

*a* = 8.8115 (18) Å
*b* = 7.7224 (16) Å
*c* = 11.997 (3) Å
β = 104.246 (4)°
*V* = 791.3 (3) Å^3^
*Z* = 4
Mo *K*α radiation
μ = 19.03 mm^−1^
*T* = 297 (2) K
0.23 × 0.21 × 0.19 mm

#### Data collection

Bruker SMART APEX CCD diffractometer
Absorption correction: numerical (*SADABS*; Sheldrick, 2003 ▶) *T*_min_ = 0.118, *T*_max_ = 0.429
8611 measured reflections
1568 independent reflections
1151 reflections with *I* > 2σ(*I*)
*R*_int_ = 0.093

#### Refinement

*R*[*F* ^2^ > 2σ(*F* ^2^)] = 0.037
*wR*(*F* ^2^) = 0.091
*S* = 1.05
1568 reflections
118 parameters
Δρ_max_ = 1.28 e Å^−3^
Δρ_min_ = −0.90 e Å^−3^

### 

Data collection: *SMART* (Bruker, 2005 ▶); cell refinement: *SAINT-Plus* (Bruker, 2003 ▶); data reduction: *SAINT-Plus*; program(s) used to solve structure: *SHELXTL* (Sheldrick, 2008 ▶); program(s) used to refine structure: *SHELXTL*; molecular graphics: *DIAMOND* (Brandenburg & Putz, 2007 ▶); software used to prepare material for publication: *SHELXTL*.

## Supplementary Material

Crystal structure: contains datablocks global, I. DOI: 10.1107/S1600536808005783/mg2048sup1.cif

Structure factors: contains datablocks I. DOI: 10.1107/S1600536808005783/mg2048Isup2.hkl

Additional supplementary materials:  crystallographic information; 3D view; checkCIF report

## Figures and Tables

**Table:** 

Ga1—O3^i^	1.834 (5)
Ga1—O4^ii^	1.834 (5)
Ga1—O6	1.831 (5)
Ga1—O7^iii^	1.790 (5)
Ga2—O1	1.840 (5)
Ga2—O2^iii^	1.838 (5)
Ga2—O5^iv^	1.832 (5)
Ga2—O7	1.810 (5)
B1—O1	1.376 (10)
B1—O2^v^	1.370 (10)
B1—O3	1.358 (10)
B2—O4	1.366 (9)
B2—O5	1.395 (9)
B2—O6^iii^	1.341 (10)

**Table:** 

O7^iii^—Ga1—O6	110.8 (2)
O7^iii^—Ga1—O4^ii^	110.4 (2)
O6—Ga1—O4^ii^	114.5 (2)
O7^iii^—Ga1—O3^i^	110.4 (2)
O6—Ga1—O3^i^	105.7 (2)
O4^ii^—Ga1—O3^i^	104.8 (2)
O7—Ga2—O5^iv^	109.3 (2)
O7—Ga2—O2^iii^	109.5 (2)
O5^iv^—Ga2—O2^iii^	110.6 (2)
O7—Ga2—O1	112.5 (2)
O5^iv^—Ga2—O1	109.3 (2)
O2^iii^—Ga2—O1	105.7 (2)
O3—B1—O2^v^	119.4 (7)
O3—B1—O1	117.8 (8)
O2^v^—B1—O1	122.7 (7)
O6^iii^—B2—O4	124.7 (7)
O6^iii^—B2—O5	116.3 (7)
O4—B2—O5	119.0 (7)

Symmetry codes: (i) 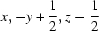; (ii) 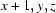; (iii) 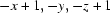; (iv) 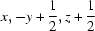; (v) 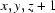.

**Fig. 1.**
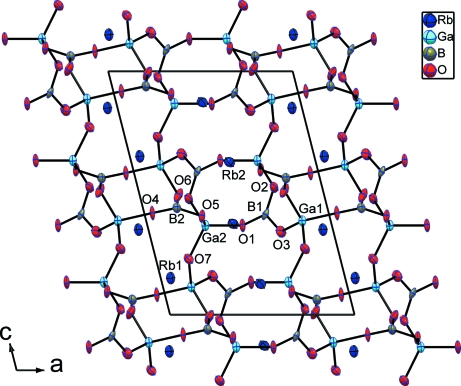
View of the unit cell along the b axis. Displacement ellipsoids are drawn at the 50% probability level.

## supplementary crystallographic information

### Comment

Complex metal borates adopt various structure types that result from the many possible geometric arrangements formed by metal-centered polyhedra and borate anions, which can be either three- or four-coordinate. They are also of interest as nonlinear optical materials, such as β-BaB_2_O_4_, LiB_3_O_5_, and YAl_3_(BO_3_)_4_ (Chen *et al.*, 2004). For these reasons, we have examined the phase diagrams of alkali metal gallium borates and have determined the crystal structures of some of the materials discovered. The homologous series A_2_Ga_2_O(BO_3_)_2_ (A = Na, K, Rb, Cs) is a portion of the new compounds discovered to date. In each, pairs of gallium-centered tetrahedra are connected through a shared oxygen vertex, and the tetrahedral basal planes are connected through shared oxygen vertices with triangular orthoborate anions. Depending on the size of the alkali metal ions, which occupy channels or spaces within the three-dimensional network, the compounds crystallize in different space groups: *P*31*c* for the Na member (Corbel & Leblanc, 2000), P321 for the K member (Smith *et al.*, 1997), and *P*2_1_/*c* for the Cs member (Smith, 1995), which is isotypic with the Rb compound reported herein (Fig. 1).

### Experimental

Powders of Rb_2_Ga_2_O(BO_3_)_2_ were prepared from stoichiometric mixtures of RbNO_3_, Ga(NO_3_)_3_, and H_3_BO_3_, which were decomposed in alumina crucibles at 300 °C and then heated to 500 °C at 50 °C increments, with a soak of several hours at each temperature and intermediate grinding between each soak period. Crystals were grown in a platinum dish from a 1:1 molar mixture of the prepared powder in the presence of Rb_3_BO_3_ flux. The mixture was heated to 700 °C and cooled at 10 °C/hour to room temperature, and a single-crystal was cut from the crystal mass for subsequent X-ray diffraction analysis.

### Refinement

The highest peak and the deepest hole are located 0.74 Å and 1.13 Å, respectively, from Rb2.

### Crystal data

**Table:**

Rb_2_Ga_2_O(BO_3_)_2_	*F*_000_ = 808
*M**_r_* = 444.00	*D*_x_ = 3.727 Mg m^−^^3^
Monoclinic, *P*2_1_/*c*	Mo *K*α radiation λ = 0.71073 Å
Hall symbol: -P 2ybc	Cell parameters from 1660 reflections
*a* = 8.8115 (18) Å	θ = 3.7–25.7º
*b* = 7.7224 (16) Å	µ = 19.03 mm^−^^1^
*c* = 11.997 (3) Å	*T* = 297 (2) K
β = 104.246 (4)º	Block, colorless
*V* = 791.3 (3) Å^3^	0.23 × 0.21 × 0.19 mm
*Z* = 4	

### Data collection

**Table:**

Bruker SMART APEX CCD diffractometer	1568 independent reflections
Radiation source: fine-focus sealed tube	1151 reflections with *I* > 2σ(*I*)
Monochromator: graphite	*R*_int_ = 0.093
*T* = 297(2) K	θ_max_ = 26.1º
ω scans	θ_min_ = 2.4º
Absorption correction: numerical(SADABS; Sheldrick, 2003)	*h* = −10→10
*T*_min_ = 0.118, *T*_max_ = 0.429	*k* = −9→9
8611 measured reflections	*l* = −14→14

### Refinement

**Table:**

Refinement on *F*^2^	Primary atom site location: structure-invariant direct methods
Least-squares matrix: full	Secondary atom site location: difference Fourier map
*R*[*F*^2^ > 2σ(*F*^2^)] = 0.037	*w* = 1/[σ^2^(*F*_o_^2^) + (0.0376*P*)^2^] where *P* = (*F*_o_^2^ + 2*F*_c_^2^)/3
*wR*(*F*^2^) = 0.091	(Δ/σ)_max_ < 0.001
*S* = 1.05	Δρ_max_ = 1.28 e Å^−^^3^
1568 reflections	Δρ_min_ = −0.90 e Å^−^^3^
118 parameters	Extinction correction: none

### Special details

**Table:**

Geometry. All e.s.d.'s (except the e.s.d. in the dihedral angle between two l.s. planes) are estimated using the full covariance matrix. The cell e.s.d.'s are taken into account individually in the estimation of e.s.d.'s in distances, angles and torsion angles; correlations between e.s.d.'s in cell parameters are only used when they are defined by crystal symmetry. An approximate (isotropic) treatment of cell e.s.d.'s is used for estimating e.s.d.'s involving l.s. planes.
Refinement. Refinement of *F*^2^ against ALL reflections. The weighted *R*-factor *wR* and goodness of fit *S* are based on *F*^2^, conventional *R*-factors *R* are based on *F*, with *F* set to zero for negative *F*^2^. The threshold expression of *F*^2^ > σ(*F*^2^) is used only for calculating *R*-factors(gt) *etc*. and is not relevant to the choice of reflections for refinement. *R*-factors based on *F*^2^ are statistically about twice as large as those based on *F*, and *R*- factors based on ALL data will be even larger.

### Fractional atomic coordinates and isotropic or equivalent isotropic displacement parameters (Å^2^)

**Table:**

	*x*	*y*	*z*	*U*_iso_*/*U*_eq_	
Rb1	0.05407 (9)	0.12523 (11)	0.15061 (7)	0.0276 (2)	
Rb2	0.53288 (9)	0.12728 (10)	0.62677 (7)	0.0265 (2)	
Ga1	0.84630 (9)	0.12201 (10)	0.38547 (7)	0.0183 (2)	
Ga2	0.31410 (9)	0.06933 (11)	0.86673 (8)	0.0191 (2)	
B1	0.6554 (10)	0.0915 (10)	0.9123 (8)	0.0166 (19)	
B2	0.1834 (10)	0.1269 (11)	0.4388 (8)	0.0181 (18)	
O1	0.5159 (5)	0.0083 (6)	0.8667 (5)	0.0221 (13)	
O2	0.7394 (6)	0.0677 (7)	0.0235 (4)	0.0249 (13)	
O3	0.7155 (6)	0.1924 (7)	0.8410 (5)	0.0261 (13)	
O4	0.0442 (5)	0.2139 (6)	0.4178 (5)	0.0247 (13)	
O5	0.3091 (5)	0.2014 (6)	0.4049 (5)	0.0215 (12)	
O6	0.7902 (6)	0.0280 (7)	0.5097 (5)	0.0286 (14)	
O7	0.1781 (6)	0.0322 (7)	0.7290 (5)	0.0248 (12)	

### Atomic displacement parameters (Å^2^)

**Table:**

	*U*^11^	*U*^22^	*U*^33^	*U*^12^	*U*^13^	*U*^23^
Rb1	0.0180 (4)	0.0338 (5)	0.0310 (5)	−0.0050 (3)	0.0063 (4)	−0.0004 (4)
Rb2	0.0225 (4)	0.0282 (4)	0.0260 (5)	0.0032 (3)	0.0008 (3)	−0.0024 (4)
Ga1	0.0125 (4)	0.0193 (4)	0.0237 (5)	0.0007 (3)	0.0055 (4)	0.0004 (4)
Ga2	0.0122 (4)	0.0203 (4)	0.0252 (5)	−0.0007 (3)	0.0052 (4)	0.0005 (4)
B1	0.017 (4)	0.011 (4)	0.026 (5)	0.003 (3)	0.014 (4)	0.000 (4)
B2	0.015 (4)	0.017 (4)	0.022 (5)	0.000 (3)	0.005 (4)	0.002 (4)
O1	0.008 (3)	0.022 (3)	0.037 (4)	−0.001 (2)	0.007 (2)	−0.005 (2)
O2	0.016 (3)	0.031 (3)	0.025 (3)	−0.008 (2)	0.002 (2)	0.010 (3)
O3	0.024 (3)	0.024 (3)	0.029 (3)	−0.012 (2)	0.004 (3)	0.000 (3)
O4	0.008 (3)	0.023 (3)	0.043 (4)	−0.006 (2)	0.008 (2)	0.001 (3)
O5	0.011 (3)	0.021 (3)	0.035 (3)	0.001 (2)	0.011 (2)	0.004 (2)
O6	0.029 (3)	0.024 (3)	0.040 (4)	0.004 (2)	0.023 (3)	0.010 (3)
O7	0.019 (3)	0.026 (3)	0.026 (3)	0.003 (2)	−0.001 (2)	−0.006 (3)

### Geometric parameters (Å, °)

**Table:**

Rb1—O2^i^	2.851 (5)	Ga2—O2^iv^	1.838 (5)
Rb1—O7^ii^	2.928 (5)	Ga2—O5^viii^	1.832 (5)
Rb1—O7^iii^	3.034 (5)	Ga2—O7	1.810 (5)
Rb1—O4^ii^	3.039 (5)	Ga2—Rb1^iii^	3.5346 (12)
Rb1—O3^iv^	3.170 (5)	Ga2—Rb2^xiii^	3.6641 (13)
Rb1—B1^iv^	3.298 (8)	Ga2—Rb1^viii^	3.8226 (12)
Rb1—O4	3.300 (6)	Ga2—Rb2^viii^	3.9973 (13)
Rb1—O4^v^	3.341 (5)	B1—O1	1.376 (10)
Rb1—B2	3.363 (9)	B1—O2^xiv^	1.370 (10)
Rb1—O5	3.365 (5)	B1—O3	1.358 (10)
Rb1—O2^vi^	3.430 (6)	B1—Rb1^iv^	3.298 (8)
Rb1—Ga2^iii^	3.5346 (12)	B1—Rb2^viii^	3.724 (8)
Rb2—O3	2.722 (5)	B2—O4	1.366 (9)
Rb2—O5	2.953 (5)	B2—O5	1.395 (9)
Rb2—O5^iv^	2.964 (5)	B2—O6^iv^	1.341 (10)
Rb2—O1^vii^	2.977 (5)	B2—Rb2^iv^	3.419 (8)
Rb2—O6	3.045 (5)	B2—Rb1^viii^	3.584 (9)
Rb2—O1	3.060 (5)	O1—Rb2^xiii^	2.977 (5)
Rb2—O6^iv^	3.151 (6)	O2—B1^xv^	1.370 (10)
Rb2—B2	3.340 (9)	O2—Ga2^iv^	1.838 (5)
Rb2—B1	3.340 (9)	O2—Rb1^ix^	2.851 (5)
Rb2—O2^viii^	3.392 (6)	O2—Rb2^ii^	3.392 (6)
Rb2—B2^iv^	3.419 (8)	O2—Rb1^vi^	3.430 (6)
Rb2—Rb2^iv^	3.5477 (17)	O3—Ga1^viii^	1.834 (5)
Ga1—O3^ii^	1.834 (5)	O3—Rb1^iv^	3.170 (5)
Ga1—O4^ix^	1.834 (5)	O4—Ga1^i^	1.834 (5)
Ga1—O6	1.831 (5)	O4—Rb1^viii^	3.039 (5)
Ga1—O7^iv^	1.790 (5)	O4—Rb1^xvi^	3.341 (5)
Ga1—Rb1^ix^	3.7189 (13)	O5—Ga2^ii^	1.832 (5)
Ga1—Rb1^x^	3.7966 (13)	O5—Rb2^iv^	2.964 (5)
Ga1—Rb2^iv^	3.8275 (13)	O6—B2^iv^	1.341 (10)
Ga1—Rb1^xi^	3.9828 (14)	O6—Rb2^iv^	3.151 (6)
Ga1—Rb1^xii^	4.0307 (14)	O7—Ga1^iv^	1.790 (5)
Ga1—Rb2^ii^	4.0975 (12)	O7—Rb1^viii^	2.928 (5)
Ga2—O1	1.840 (5)	O7—Rb1^iii^	3.034 (5)
			
O2^i^—Rb1—O7^ii^	123.34 (15)	Rb1^x^—Ga1—Rb2^iv^	121.81 (3)
O2^i^—Rb1—O7^iii^	60.74 (14)	O7^iv^—Ga1—Rb1^xi^	42.60 (17)
O7^ii^—Rb1—O7^iii^	116.83 (11)	O6—Ga1—Rb1^xi^	79.48 (16)
O2^i^—Rb1—O4^ii^	76.60 (14)	O4^ix^—Ga1—Rb1^xi^	99.91 (16)
O7^ii^—Rb1—O4^ii^	81.35 (14)	O3^ii^—Ga1—Rb1^xi^	149.37 (17)
O7^iii^—Rb1—O4^ii^	137.01 (13)	Rb1^ix^—Ga1—Rb1^xi^	76.19 (2)
O2^i^—Rb1—O3^iv^	115.30 (15)	Rb1^x^—Ga1—Rb1^xi^	120.83 (2)
O7^ii^—Rb1—O3^iv^	120.23 (13)	Rb2^iv^—Ga1—Rb1^xi^	74.04 (2)
O7^iii^—Rb1—O3^iv^	100.16 (13)	O7^iv^—Ga1—Rb1^xii^	123.18 (18)
O4^ii^—Rb1—O3^iv^	102.65 (13)	O6—Ga1—Rb1^xii^	125.38 (17)
O2^i^—Rb1—B1^iv^	120.40 (19)	O4^ix^—Ga1—Rb1^xii^	55.16 (16)
O7^ii^—Rb1—B1^iv^	106.42 (17)	O3^ii^—Ga1—Rb1^xii^	49.70 (17)
O7^iii^—Rb1—B1^iv^	123.92 (17)	Rb1^ix^—Ga1—Rb1^xii^	75.60 (2)
O4^ii^—Rb1—B1^iv^	81.11 (18)	Rb1^x^—Ga1—Rb1^xii^	61.41 (3)
O3^iv^—Rb1—B1^iv^	24.13 (17)	Rb2^iv^—Ga1—Rb1^xii^	133.78 (3)
O2^i^—Rb1—O4	107.29 (14)	Rb1^xi^—Ga1—Rb1^xii^	149.02 (3)
O7^ii^—Rb1—O4	66.27 (13)	O7^iv^—Ga1—Rb2^ii^	78.58 (17)
O7^iii^—Rb1—O4	55.84 (13)	O6—Ga1—Rb2^ii^	122.49 (17)
O4^ii^—Rb1—O4	143.75 (4)	O4^ix^—Ga1—Rb2^ii^	113.96 (17)
O3^iv^—Rb1—O4	107.42 (13)	O3^ii^—Ga1—Rb2^ii^	32.03 (17)
B1^iv^—Rb1—O4	122.35 (19)	Rb1^ix^—Ga1—Rb2^ii^	77.80 (3)
O2^i^—Rb1—O4^v^	63.98 (14)	Rb1^x^—Ga1—Rb2^ii^	119.20 (3)
O7^ii^—Rb1—O4^v^	172.59 (13)	Rb2^iv^—Ga1—Rb2^ii^	77.84 (2)
O7^iii^—Rb1—O4^v^	64.64 (13)	Rb1^xi^—Ga1—Rb2^ii^	119.89 (3)
O4^ii^—Rb1—O4^v^	102.47 (12)	Rb1^xii^—Ga1—Rb2^ii^	65.61 (2)
O3^iv^—Rb1—O4^v^	52.94 (12)	O7—Ga2—O5^viii^	109.3 (2)
B1^iv^—Rb1—O4^v^	68.29 (16)	O7—Ga2—O2^iv^	109.5 (2)
O4—Rb1—O4^v^	111.63 (12)	O5^viii^—Ga2—O2^iv^	110.6 (2)
O2^i^—Rb1—B2	126.09 (18)	O7—Ga2—O1	112.5 (2)
O7^ii^—Rb1—B2	70.20 (18)	O5^viii^—Ga2—O1	109.3 (2)
O7^iii^—Rb1—B2	67.02 (17)	O2^iv^—Ga2—O1	105.7 (2)
O4^ii^—Rb1—B2	150.35 (17)	O7—Ga2—Rb1^iii^	59.13 (17)
O3^iv^—Rb1—B2	85.29 (17)	O5^viii^—Ga2—Rb1^iii^	110.32 (14)
B1^iv^—Rb1—B2	98.7 (2)	O2^iv^—Ga2—Rb1^iii^	53.43 (16)
O4—Rb1—B2	23.63 (16)	O1—Ga2—Rb1^iii^	139.84 (15)
O4^v^—Rb1—B2	104.94 (17)	O7—Ga2—Rb2^xiii^	91.67 (16)
O2^i^—Rb1—O5	148.92 (14)	O5^viii^—Ga2—Rb2^xiii^	157.90 (16)
O7^ii^—Rb1—O5	55.79 (13)	O2^iv^—Ga2—Rb2^xiii^	66.83 (17)
O7^iii^—Rb1—O5	90.94 (13)	O1—Ga2—Rb2^xiii^	53.89 (15)
O4^ii^—Rb1—O5	128.56 (13)	Rb1^iii^—Ga2—Rb2^xiii^	86.15 (3)
O3^iv^—Rb1—O5	80.03 (13)	O7—Ga2—Rb1^viii^	47.68 (16)
B1^iv^—Rb1—O5	85.03 (18)	O5^viii^—Ga2—Rb1^viii^	61.69 (16)
O4—Rb1—O5	41.80 (11)	O2^iv^—Ga2—Rb1^viii^	128.63 (16)
O4^v^—Rb1—O5	117.62 (12)	O1—Ga2—Rb1^viii^	125.18 (17)
B2—Rb1—O5	23.93 (16)	Rb1^iii^—Ga2—Rb1^viii^	80.45 (2)
O2^i^—Rb1—O2^vi^	101.45 (12)	Rb2^xiii^—Ga2—Rb1^viii^	138.19 (3)
O7^ii^—Rb1—O2^vi^	112.08 (14)	O7—Ga2—Rb2	71.19 (17)
O7^iii^—Rb1—O2^vi^	129.57 (13)	O5^viii^—Ga2—Rb2	96.97 (15)
O4^ii^—Rb1—O2^vi^	61.13 (12)	O2^iv^—Ga2—Rb2	149.64 (18)
O3^iv^—Rb1—O2^vi^	41.56 (13)	O1—Ga2—Rb2	50.97 (17)
B1^iv^—Rb1—O2^vi^	23.39 (18)	Rb1^iii^—Ga2—Rb2	128.63 (3)
O4—Rb1—O2^vi^	145.74 (12)	Rb2^xiii^—Ga2—Rb2	82.84 (2)
O4^v^—Rb1—O2^vi^	65.28 (12)	Rb1^viii^—Ga2—Rb2	75.46 (3)
B2—Rb1—O2^vi^	122.11 (17)	O7—Ga2—Rb2^viii^	152.81 (16)
O5—Rb1—O2^vi^	106.98 (12)	O5^viii^—Ga2—Rb2^viii^	43.56 (16)
O2^i^—Rb1—Ga2^iii^	31.19 (10)	O2^iv^—Ga2—Rb2^viii^	86.43 (17)
O7^ii^—Rb1—Ga2^iii^	131.89 (11)	O1—Ga2—Rb2^viii^	82.52 (16)
O7^iii^—Rb1—Ga2^iii^	30.80 (10)	Rb1^iii^—Ga2—Rb2^viii^	123.67 (3)
O4^ii^—Rb1—Ga2^iii^	107.55 (9)	Rb2^xiii^—Ga2—Rb2^viii^	115.16 (3)
O3^iv^—Rb1—Ga2^iii^	104.14 (9)	Rb1^viii^—Ga2—Rb2^viii^	105.14 (3)
B1^iv^—Rb1—Ga2^iii^	121.58 (14)	Rb2—Ga2—Rb2^viii^	106.28 (3)
O4—Rb1—Ga2^iii^	84.39 (8)	O3—B1—O2^xiv^	119.4 (7)
O4^v^—Rb1—Ga2^iii^	53.33 (8)	O3—B1—O1	117.8 (8)
B2—Rb1—Ga2^iii^	97.81 (14)	O2^xiv^—B1—O1	122.7 (7)
O5—Rb1—Ga2^iii^	121.74 (8)	O3—B1—Rb1^iv^	72.7 (4)
O2^vi^—Rb1—Ga2^iii^	113.35 (9)	O2^xiv^—B1—Rb1^iv^	83.7 (4)
O3—Rb2—O5	157.47 (16)	O1—B1—Rb1^iv^	109.9 (4)
O3—Rb2—O5^iv^	95.27 (15)	O3—B1—Rb2	52.0 (4)
O5—Rb2—O5^iv^	106.33 (12)	O2^xiv^—B1—Rb2	166.2 (5)
O3—Rb2—O1^vii^	81.27 (15)	O1—B1—Rb2	66.4 (4)
O5—Rb2—O1^vii^	76.31 (14)	Rb1^iv^—B1—Rb2	83.14 (19)
O5^iv^—Rb2—O1^vii^	157.67 (13)	O3—B1—Rb2^viii^	109.0 (5)
O3—Rb2—O6	98.49 (16)	O2^xiv^—B1—Rb2^viii^	65.4 (4)
O5—Rb2—O6	92.34 (14)	O1—B1—Rb2^viii^	99.5 (4)
O5^iv^—Rb2—O6	45.46 (13)	Rb1^iv^—B1—Rb2^viii^	145.8 (3)
O1^vii^—Rb2—O6	112.93 (14)	Rb2—B1—Rb2^viii^	125.8 (2)
O3—Rb2—O1	47.31 (13)	O6^iv^—B2—O4	124.7 (7)
O5—Rb2—O1	136.88 (12)	O6^iv^—B2—O5	116.3 (7)
O5^iv^—Rb2—O1	89.80 (13)	O4—B2—O5	119.0 (7)
O1^vii^—Rb2—O1	103.37 (11)	O6^iv^—B2—Rb2	70.3 (4)
O6—Rb2—O1	124.94 (13)	O4—B2—Rb2	139.5 (5)
O3—Rb2—O6^iv^	144.03 (14)	O5—B2—Rb2	62.0 (4)
O5—Rb2—O6^iv^	44.59 (13)	O6^iv^—B2—Rb1	116.6 (5)
O5^iv^—Rb2—O6^iv^	90.03 (13)	O4—B2—Rb1	75.6 (4)
O1^vii^—Rb2—O6^iv^	105.77 (13)	O5—B2—Rb1	78.1 (4)
O6—Rb2—O6^iv^	110.17 (11)	Rb2—B2—Rb1	135.8 (3)
O1—Rb2—O6^iv^	97.31 (13)	O6^iv^—B2—Rb2^iv^	62.6 (4)
O3—Rb2—B2	150.50 (19)	O4—B2—Rb2^iv^	156.1 (6)
O5—Rb2—B2	24.64 (17)	O5—B2—Rb2^iv^	59.4 (4)
O5^iv^—Rb2—B2	107.28 (18)	Rb2—B2—Rb2^iv^	63.31 (15)
O1^vii^—Rb2—B2	84.46 (17)	Rb1—B2—Rb2^iv^	80.99 (19)
O6—Rb2—B2	110.85 (19)	O6^iv^—B2—Rb1^viii^	101.5 (5)
O1—Rb2—B2	112.62 (17)	O4—B2—Rb1^viii^	56.0 (4)
O6^iv^—Rb2—B2	23.61 (16)	O5—B2—Rb1^viii^	113.6 (5)
O3—Rb2—B1	23.13 (17)	Rb2—B2—Rb1^viii^	85.5 (2)
O5—Rb2—B1	156.39 (16)	Rb1—B2—Rb1^viii^	130.1 (2)
O5^iv^—Rb2—B1	90.97 (16)	Rb2^iv^—B2—Rb1^viii^	148.0 (3)
O1^vii^—Rb2—B1	93.85 (17)	B1—O1—Ga2	130.5 (5)
O6—Rb2—B1	111.28 (17)	B1—O1—Rb2^xiii^	124.9 (4)
O1—Rb2—B1	24.32 (16)	Ga2—O1—Rb2^xiii^	96.17 (18)
O6^iv^—Rb2—B1	121.60 (17)	B1—O1—Rb2	89.3 (4)
B2—Rb2—B1	134.8 (2)	Ga2—O1—Rb2	101.2 (2)
O3—Rb2—O2^viii^	88.32 (14)	Rb2^xiii^—O1—Rb2	111.24 (16)
O5—Rb2—O2^viii^	80.53 (13)	B1^xv^—O2—Ga2^iv^	127.1 (5)
O5^iv^—Rb2—O2^viii^	103.70 (12)	B1^xv^—O2—Rb1^ix^	136.1 (4)
O1^vii^—Rb2—O2^viii^	54.35 (12)	Ga2^iv^—O2—Rb1^ix^	95.4 (2)
O6—Rb2—O2^viii^	58.60 (12)	B1^xv^—O2—Rb2^ii^	93.0 (4)
O1—Rb2—O2^viii^	134.98 (13)	Ga2^iv^—O2—Rb2^ii^	83.28 (19)
O6^iv^—Rb2—O2^viii^	124.89 (13)	Rb1^ix^—O2—Rb2^ii^	103.59 (16)
B2—Rb2—O2^viii^	104.03 (17)	B1^xv^—O2—Rb1^vi^	72.9 (4)
B1—Rb2—O2^viii^	111.40 (16)	Ga2^iv^—O2—Rb1^vi^	117.0 (2)
O3—Rb2—B2^iv^	91.56 (19)	Rb1^ix^—O2—Rb1^vi^	78.55 (12)
O5—Rb2—B2^iv^	105.54 (18)	Rb2^ii^—O2—Rb1^vi^	159.51 (17)
O5^iv^—Rb2—B2^iv^	23.90 (16)	B1—O3—Ga1^viii^	125.4 (5)
O1^vii^—Rb2—B2^iv^	133.78 (17)	B1—O3—Rb2	104.9 (5)
O6—Rb2—B2^iv^	23.01 (17)	Ga1^viii^—O3—Rb2	127.0 (2)
O1—Rb2—B2^iv^	104.57 (17)	B1—O3—Rb1^iv^	83.2 (4)
O6^iv^—Rb2—B2^iv^	106.29 (17)	Ga1^viii^—O3—Rb1^iv^	104.1 (2)
B2—Rb2—B2^iv^	116.69 (15)	Rb2—O3—Rb1^iv^	96.45 (16)
B1—Rb2—B2^iv^	96.8 (2)	B2—O4—Ga1^i^	127.8 (5)
O2^viii^—Rb2—B2^iv^	80.00 (16)	B2—O4—Rb1^viii^	102.1 (5)
O3—Rb2—Rb2^iv^	147.74 (12)	Ga1^i^—O4—Rb1^viii^	99.4 (2)
O5—Rb2—Rb2^iv^	53.31 (10)	B2—O4—Rb1	80.8 (4)
O5^iv^—Rb2—Rb2^iv^	53.02 (10)	Ga1^i^—O4—Rb1	88.01 (19)
O1^vii^—Rb2—Rb2^iv^	125.17 (11)	Rb1^viii^—O4—Rb1	167.46 (17)
O6—Rb2—Rb2^iv^	56.49 (11)	B2—O4—Rb1^xvi^	132.8 (4)
O1—Rb2—Rb2^iv^	127.20 (10)	Ga1^i^—O4—Rb1^xvi^	98.07 (19)
O6^iv^—Rb2—Rb2^iv^	53.68 (10)	Rb1^viii^—O4—Rb1^xvi^	77.53 (12)
B2—Rb2—Rb2^iv^	59.44 (15)	Rb1—O4—Rb1^xvi^	91.48 (13)
B1—Rb2—Rb2^iv^	140.98 (13)	B2—O5—Ga2^ii^	122.8 (5)
O2^viii^—Rb2—Rb2^iv^	93.49 (9)	B2—O5—Rb2	93.4 (4)
B2^iv^—Rb2—Rb2^iv^	57.25 (15)	Ga2^ii^—O5—Rb2	111.1 (2)
O7^iv^—Ga1—O6	110.8 (2)	B2—O5—Rb2^iv^	96.7 (4)
O7^iv^—Ga1—O4^ix^	110.4 (2)	Ga2^ii^—O5—Rb2^iv^	139.0 (2)
O6—Ga1—O4^ix^	114.5 (2)	Rb2—O5—Rb2^iv^	73.67 (11)
O7^iv^—Ga1—O3^ii^	110.4 (2)	B2—O5—Rb1	77.9 (4)
O6—Ga1—O3^ii^	105.7 (2)	Ga2^ii^—O5—Rb1	89.68 (19)
O4^ix^—Ga1—O3^ii^	104.8 (2)	Rb2—O5—Rb1	158.76 (17)
O7^iv^—Ga1—Rb1^ix^	53.98 (17)	Rb2^iv^—O5—Rb1	87.95 (13)
O6—Ga1—Rb1^ix^	154.26 (16)	B2^iv^—O6—Ga1	132.7 (5)
O4^ix^—Ga1—Rb1^ix^	62.46 (17)	B2^iv^—O6—Rb2	94.4 (4)
O3^ii^—Ga1—Rb1^ix^	99.52 (17)	Ga1—O6—Rb2	131.0 (2)
O7^iv^—Ga1—Rb1^x^	157.95 (16)	B2^iv^—O6—Rb2^iv^	86.1 (4)
O6—Ga1—Rb1^x^	72.13 (18)	Ga1—O6—Rb2^iv^	96.8 (2)
O4^ix^—Ga1—Rb1^x^	52.16 (17)	Rb2—O6—Rb2^iv^	69.83 (11)
O3^ii^—Ga1—Rb1^x^	88.92 (17)	Ga1^iv^—O7—Ga2	137.2 (3)
Rb1^ix^—Ga1—Rb1^x^	113.93 (3)	Ga1^iv^—O7—Rb1^viii^	113.0 (2)
O7^iv^—Ga1—Rb2^iv^	72.36 (17)	Ga2—O7—Rb1^viii^	105.1 (2)
O6—Ga1—Rb2^iv^	54.84 (18)	Ga1^iv^—O7—Rb1^iii^	97.5 (2)
O4^ix^—Ga1—Rb2^iv^	168.11 (17)	Ga2—O7—Rb1^iii^	90.1 (2)
O3^ii^—Ga1—Rb2^iv^	84.37 (17)	Rb1^viii^—O7—Rb1^iii^	105.83 (16)
Rb1^ix^—Ga1—Rb2^iv^	124.20 (3)		

Symmetry codes: (i) *x*−1, *y*, *z*; (ii) *x*, −*y*+1/2, *z*−1/2; (iii) −*x*, −*y*, −*z*+1; (iv) −*x*+1, −*y*, −*z*+1; (v) −*x*, *y*−1/2, −*z*+1/2; (vi) −*x*+1, −*y*, −*z*; (vii) −*x*+1, *y*+1/2, −*z*+3/2; (viii) *x*, −*y*+1/2, *z*+1/2; (ix) *x*+1, *y*, *z*; (x) *x*+1, −*y*+1/2, *z*+1/2; (xi) −*x*+1, *y*−1/2, −*z*+1/2; (xii) −*x*+1, *y*+1/2, −*z*+1/2; (xiii) −*x*+1, *y*−1/2, −*z*+3/2; (xiv) *x*, *y*, *z*+1; (xv) *x*, *y*, *z*−1; (xvi) −*x*, *y*+1/2, −*z*+1/2.
